# 3-Anilino-1,3-di-2-pyridylpropan-1-one

**DOI:** 10.1107/S160053680904121X

**Published:** 2009-10-17

**Authors:** Hai-Xing Liu

**Affiliations:** a Microscale Science Institute, Department of Chemistry and Chemical Engineering, Weifang University, Weifang 261061, People’s Republic of China

## Abstract

The title compound, C_19_H_17_N_3_O, was prepared by the 1,4-addition reaction of 1,3-di-2-pyridylprop-2-en-1-one with aniline, and includes one chiral C atom of the methine group with an *R* configuration. The crystal structure is stabilized by inter­molecular N—H⋯N and C—H⋯O hydrogen bonds. The crystal structure also exhibits weak inter­molecular C—H⋯π inter­actions between a pyridyl H atom and the phenyl ring of adjacent mol­ecules.

## Related literature

For properties of binucleating ligand coordination compounds, see: Casalino *et al.* (2009 ▶); Clare *et al.* (2004 ▶); Lam *et al.* (1996 ▶). For multiple pyridyl compounds, see: Huang *et al.* (2008 ▶). For related structures, see: Champouret *et al.* (2006 ▶); Murthy *et al.* (2001 ▶).
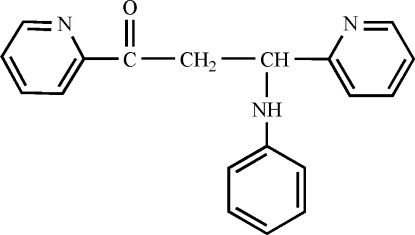


## Experimental

### 

#### Crystal data

C_19_H_17_N_3_O
*M* *_r_* = 303.36
Orthorhombic, 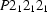

*a* = 9.316 (2) Å
*b* = 10.275 (2) Å
*c* = 16.652 (3) Å
*V* = 1594.0 (5) Å^3^
*Z* = 4
Mo *K*α radiation
μ = 0.08 mm^−1^
*T* = 293 K
0.35 × 0.30 × 0.24 mm

#### Data collection

Bruker SMART CCD diffractometer
Absorption correction: multi-scan (*SADABS*; Sheldrick (2000 ▶) *T*_min_ = 0.950, *T*_max_ = 0.976
7562 measured reflections
1961 independent reflections
1040 reflections with *I* > 2σ(*I*)
*R*_int_ = 0.077

#### Refinement

*R*[*F* ^2^ > 2σ(*F* ^2^)] = 0.048
*wR*(*F* ^2^) = 0.118
*S* = 1.00
1961 reflections
208 parameters
H-atom parameters constrained
Δρ_max_ = 0.15 e Å^−3^
Δρ_min_ = −0.13 e Å^−3^

### 

Data collection: *SMART* (Bruker, 1997 ▶); cell refinement: *SAINT* (Bruker, 1997 ▶); data reduction: *SAINT*; program(s) used to solve structure: *SHELXS97* (Sheldrick, 2008 ▶); program(s) used to refine structure: *SHELXL97* (Sheldrick, 2008 ▶); molecular graphics: *SHELXTL* (Sheldrick, 2008 ▶); software used to prepare material for publication: *SHELXTL*.

## Supplementary Material

Crystal structure: contains datablocks global, I. DOI: 10.1107/S160053680904121X/lx2115sup1.cif

Structure factors: contains datablocks I. DOI: 10.1107/S160053680904121X/lx2115Isup2.hkl

Additional supplementary materials:  crystallographic information; 3D view; checkCIF report

## Figures and Tables

**Table 1:** Hydrogen-bond geometry (Å, °)

*D*—H⋯*A*	*D*—H	H⋯*A*	*D*⋯*A*	*D*—H⋯*A*
N3—H3*N*⋯N2^i^	0.86	2.35	3.191 (4)	164
C10—H10⋯O1^i^	0.93	2.62	3.398 (5)	141
C3—H3⋯*Cg*^ii^	0.93	2.77	3.548 (5)	142

Symmetry codes: (i) 
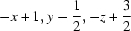
; (ii) 
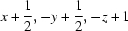
. *Cg* is the centroid of the C14–C19 phenyl ring.

**Fig. 1.**
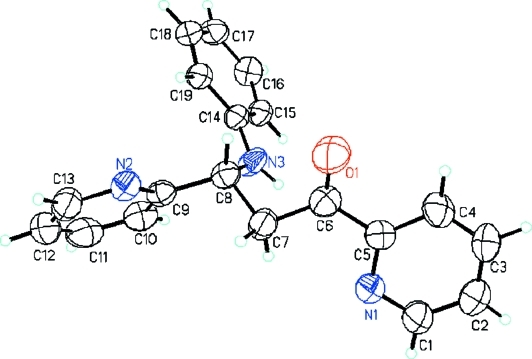
The molecular structure of the title compound with the atom numbering scheme. Displacement ellipsoids are drawn at the 50% probability level. H atoms are presented as a small cycles of arbitrary radius.

**Fig. 2.**
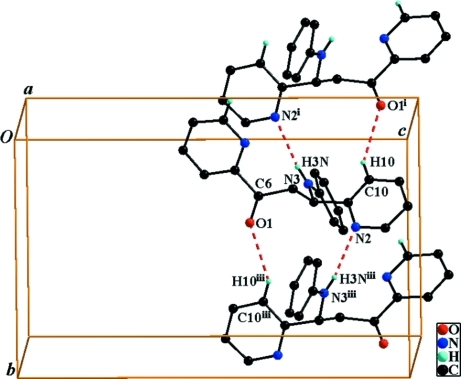
C—H···O and N—H···N hydrogen bonds (dotted lines) in the title compound. [Symmetry codes: (i) - x + 1 , y - 1/2, - z + 3/2; (iii) - x + 1, y + 1/2, - z + 3/2.]

**Fig. 3.**
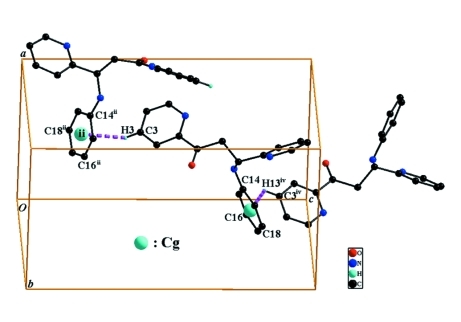
C—H···π interactions (dotted lines) in the title compound. Cg denotes the ring centroid. [Symmetry codes: (ii) x + 1/2, - y + 1/2, - z + 1; (iv) - x + 1/2, - y, z + 1/2.]

## supplementary crystallographic information

### Comment

The binucleating ligand has continued to arouse interest among chemists, because the extensive investigation of binucleating ligands plays a key role in bimetallic chemistry. These coordination compounds were potentially applied in bioinorganic chemistry, homogeneous catalysis, magnetic exchange processes, and information of performance on important enzymes (Lam *et al.*, 1996, Clare *et al.*, 2004 & Casalino *et al.*, 2009). Furthermore, compounds comprising multiple pyridyl groups are widely used in the design and self-assembly of metal-organic architectures (Huang *et al.*, 2008). Here we report the crystal structure of title compound (I) (Fig. 1).

The bond distances and angles in (I) are consistent with the values in related structures (Champouret *et al.*, 2006 & Murthy *et al.*, 2001). The chiral C8 atom possesses the expected R configuaration. The molecualr packing (Fig. 2) is stabiized by intermolecular N—H···N and C—H···O hydrogen bonds; the first between the amino H atom and the pyridyl (C9–C13/N2) N atom, with a N3—H3···N2^i^, the second between the pyridyl (C9–C13/N2) H atom and the oxygen of the C═O unit, with a C10—H10···O1^i^, respectively (Table 1). The crystal packing (Fig. 3) is further stabilized by intermolecular C—H···π interactions between the pyridyl (C1–C5/N1) H atom and the phenyl ring, with a C3—H3···Cg^ii^ (Table 1; Cg is the centroid of the C14–C19 phenyl ring).

### Experimental

1,3-di-2-pyridyl-2-en-1-one (5 mmol/1.044 g) was mixed with aniline (6 mmol/0.558 g) in toluene (20 ml). And then the phosphotungsitic (0.01 g) in water (10 ml) was added dropwise and refluxed for 2 h. The insoluble materials were produced, and then removed by filtration. The organic layer was kept at room temperature for about two days. Yellow-colored and block shaped crystals were collected (yield 67.6%).

### Refinement

All the Friedel pairs were merged. H atoms were positioned geometrically and allowed to ride on their parent atoms, with N—H and C—H distances of 0.86 and 0.93–0.96 Å, respectively, and with *U*_iso_(H) = 1.2*U*_eq_ of the parent atoms.

### Crystal data

**Table:**

C_19_H_17_N_3_O	*D*_x_ = 1.264 Mg m^−^^3^
*M**_r_* = 303.36	Melting point: 400 K
Orthorhombic, *P*2_1_2_1_2_1_	Mo *K*α radiation, λ = 0.71073 Å
Hall symbol: p 2ac 2ab	Cell parameters from 1025 reflections
*a* = 9.316 (2) Å	θ = 2.3–27.0°
*b* = 10.275 (2) Å	µ = 0.08 mm^−^^1^
*c* = 16.652 (3) Å	*T* = 293 K
*V* = 1594.0 (5) Å^3^	Block, yellow
*Z* = 4	0.35 × 0.30 × 0.24 mm
*F*(000) = 640	

### Data collection

**Table:**

Bruker SMART CCD diffractometer	1961 independent reflections
Radiation source: fine-focus sealed tube	1040 reflections with *I* > 2σ(*I*)
graphite	*R*_int_ = 0.077
Detector resolution: 10.0 pixels mm^-1^	θ_max_ = 27.0°, θ_min_ = 2.3°
φ and ω scans	*h* = −11→10
Absorption correction: multi-scan (*SADABS*; Sheldrick (2000)	*k* = −13→13
*T*_min_ = 0.950, *T*_max_ = 0.976	*l* = −13→21
7562 measured reflections	

### Refinement

**Table:**

Refinement on *F*^2^	Primary atom site location: structure-invariant direct methods
Least-squares matrix: full	Secondary atom site location: difference Fourier map
*R*[*F*^2^ > 2σ(*F*^2^)] = 0.048	Hydrogen site location: difference Fourier map
*wR*(*F*^2^) = 0.118	H-atom parameters constrained
*S* = 1.00	*w* = 1/[σ^2^(*F*_o_^2^) + (0.0005*P*)^2^ + 0.0531*P*] where *P* = (*F*_o_^2^ + 2*F*_c_^2^)/3
1961 reflections	(Δ/σ)_max_ < 0.001
208 parameters	Δρ_max_ = 0.15 e Å^−^^3^
0 restraints	Δρ_min_ = −0.13 e Å^−^^3^

### Special details

**Table:**

Geometry. All esds (except the esd in the dihedral angle between two l.s. planes) are estimated using the full covariance matrix. The cell esds are taken into account individually in the estimation of esds in distances, angles and torsion angles; correlations between esds in cell parameters are only used when they are defined by crystal symmetry. An approximate (isotropic) treatment of cell esds is used for estimating esds involving l.s. planes.
Refinement. Refinement of F^2^ against ALL reflections. The weighted R-factor wR and goodness of fit S are based on F^2^, conventional R-factors R are based on F, with F set to zero for negative F^2^. The threshold expression of F^2^ > 2sigma(F^2^) is used only for calculating R-factors(gt) etc. and is not relevant to the choice of reflections for refinement. R-factors based on F^2^ are statistically about twice as large as those based on F, and R- factors based on ALL data will be even larger.

### Fractional atomic coordinates and isotropic or equivalent isotropic displacement parameters (Å^2^)

**Table:**

	*x*	*y*	*z*	*U*_iso_*/*U*_eq_	
O1	0.5253 (4)	0.4411 (3)	0.5808 (2)	0.0934 (11)	
N1	0.6804 (4)	0.1340 (3)	0.5653 (2)	0.0659 (9)	
N2	0.6078 (3)	0.4744 (3)	0.8480 (2)	0.0551 (8)	
N3	0.3789 (3)	0.2540 (3)	0.7351 (2)	0.0628 (9)	
H3N	0.3971	0.1851	0.7076	0.075*	
C1	0.7052 (5)	0.0421 (5)	0.5109 (3)	0.0828 (14)	
H1	0.7626	−0.0277	0.5260	0.099*	
C2	0.6530 (5)	0.0426 (5)	0.4350 (3)	0.0841 (14)	
H2	0.6734	−0.0252	0.3997	0.101*	
C3	0.5700 (5)	0.1446 (5)	0.4115 (3)	0.0754 (13)	
H3	0.5324	0.1480	0.3598	0.090*	
C4	0.5428 (5)	0.2424 (4)	0.4655 (3)	0.0693 (12)	
H4	0.4868	0.3135	0.4508	0.083*	
C5	0.5998 (4)	0.2338 (3)	0.5418 (2)	0.0531 (10)	
C6	0.5753 (4)	0.3385 (4)	0.6019 (3)	0.0612 (11)	
C7	0.6147 (4)	0.3152 (4)	0.6885 (2)	0.0614 (11)	
H7A	0.6977	0.3677	0.7021	0.074*	
H7B	0.6405	0.2245	0.6956	0.074*	
C8	0.4910 (4)	0.3490 (3)	0.7454 (2)	0.0512 (10)	
H8	0.4527	0.4340	0.7293	0.061*	
C9	0.5452 (4)	0.3607 (3)	0.8309 (2)	0.0493 (9)	
C10	0.5356 (4)	0.2627 (4)	0.8858 (3)	0.0660 (11)	
H10	0.4912	0.1846	0.8726	0.079*	
C11	0.5924 (5)	0.2811 (5)	0.9609 (3)	0.0828 (14)	
H11A	0.5857	0.2156	0.9992	0.099*	
C12	0.6590 (5)	0.3961 (6)	0.9794 (3)	0.0842 (15)	
H12	0.7000	0.4101	1.0296	0.101*	
C13	0.6627 (4)	0.4886 (4)	0.9215 (3)	0.0701 (13)	
H13	0.7065	0.5674	0.9339	0.084*	
C14	0.2431 (4)	0.2689 (3)	0.7675 (2)	0.0504 (9)	
C15	0.1444 (4)	0.1682 (3)	0.7591 (2)	0.0552 (10)	
H15	0.1720	0.0907	0.7348	0.066*	
C16	0.0075 (5)	0.1827 (4)	0.7863 (3)	0.0652 (11)	
H16	−0.0574	0.1149	0.7794	0.078*	
C17	−0.0373 (4)	0.2942 (4)	0.8235 (3)	0.0654 (11)	
H17	−0.1312	0.3027	0.8417	0.078*	
C18	0.0597 (4)	0.3928 (4)	0.8330 (3)	0.0611 (11)	
H18	0.0312	0.4690	0.8586	0.073*	
C19	0.2000 (4)	0.3816 (3)	0.8054 (2)	0.0545 (10)	
H19	0.2644	0.4498	0.8124	0.065*	

### Atomic displacement parameters (Å^2^)

**Table:**

	*U*^11^	*U*^22^	*U*^33^	*U*^12^	*U*^13^	*U*^23^
O1	0.135 (3)	0.0631 (17)	0.082 (2)	0.0240 (19)	−0.001 (2)	0.0020 (17)
N1	0.071 (2)	0.067 (2)	0.060 (2)	0.0110 (19)	−0.0054 (18)	−0.008 (2)
N2	0.0545 (19)	0.0534 (19)	0.057 (2)	−0.0029 (16)	0.0051 (16)	−0.0056 (17)
N3	0.0509 (19)	0.0561 (19)	0.082 (2)	−0.0027 (16)	0.0062 (17)	−0.0321 (19)
C1	0.098 (4)	0.081 (3)	0.069 (3)	0.023 (3)	−0.011 (3)	−0.011 (3)
C2	0.096 (4)	0.083 (3)	0.073 (3)	0.008 (3)	0.002 (3)	−0.020 (3)
C3	0.094 (4)	0.082 (3)	0.050 (3)	−0.007 (3)	0.007 (2)	0.000 (3)
C4	0.084 (3)	0.066 (3)	0.057 (3)	−0.002 (2)	0.002 (2)	0.019 (2)
C5	0.055 (2)	0.047 (2)	0.057 (3)	−0.005 (2)	0.0043 (19)	0.008 (2)
C6	0.066 (3)	0.056 (2)	0.062 (3)	0.001 (2)	0.006 (2)	0.002 (2)
C7	0.064 (3)	0.065 (2)	0.055 (3)	0.002 (2)	0.005 (2)	−0.009 (2)
C8	0.050 (2)	0.0438 (19)	0.060 (3)	−0.0032 (18)	0.0016 (18)	−0.0048 (19)
C9	0.046 (2)	0.0425 (19)	0.060 (2)	0.0001 (18)	0.0037 (19)	−0.002 (2)
C10	0.068 (3)	0.052 (2)	0.077 (3)	0.001 (2)	0.009 (2)	0.012 (2)
C11	0.091 (4)	0.087 (3)	0.069 (3)	0.028 (3)	0.015 (3)	0.028 (3)
C12	0.084 (4)	0.110 (4)	0.058 (3)	0.025 (3)	−0.007 (3)	−0.008 (3)
C13	0.063 (3)	0.076 (3)	0.071 (3)	−0.001 (2)	0.002 (2)	−0.021 (3)
C14	0.052 (2)	0.046 (2)	0.054 (2)	0.0035 (18)	−0.0036 (19)	−0.007 (2)
C15	0.060 (3)	0.048 (2)	0.058 (2)	−0.001 (2)	−0.002 (2)	−0.0083 (19)
C16	0.064 (3)	0.064 (3)	0.068 (3)	−0.013 (2)	0.005 (2)	0.001 (2)
C17	0.050 (2)	0.079 (3)	0.067 (3)	0.009 (2)	0.006 (2)	0.000 (2)
C18	0.065 (3)	0.059 (2)	0.059 (3)	0.015 (2)	−0.003 (2)	−0.005 (2)
C19	0.055 (3)	0.051 (2)	0.058 (2)	0.0027 (18)	−0.006 (2)	−0.0078 (19)

### Geometric parameters (Å, °)

**Table:**

O1—C6	1.205 (4)	C8—C9	1.516 (5)
N1—C5	1.329 (4)	C8—H8	0.9800
N1—C1	1.330 (5)	C9—C10	1.363 (5)
N2—C13	1.335 (5)	C10—C11	1.370 (7)
N2—C9	1.336 (4)	C10—H10	0.9300
N3—C14	1.384 (4)	C11—C12	1.370 (6)
N3—C8	1.440 (4)	C11—H11A	0.9300
N3—H3N	0.8600	C12—C13	1.354 (6)
C1—C2	1.354 (7)	C12—H12	0.9300
C1—H1	0.9300	C13—H13	0.9300
C2—C3	1.359 (6)	C14—C19	1.379 (5)
C2—H2	0.9300	C14—C15	1.390 (5)
C3—C4	1.372 (6)	C15—C16	1.362 (5)
C3—H3	0.9300	C15—H15	0.9300
C4—C5	1.380 (6)	C16—C17	1.367 (5)
C4—H4	0.9300	C16—H16	0.9300
C5—C6	1.487 (5)	C17—C18	1.366 (5)
C6—C7	1.506 (6)	C17—H17	0.9300
C7—C8	1.531 (5)	C18—C19	1.391 (5)
C7—H7A	0.9700	C18—H18	0.9300
C7—H7B	0.9700	C19—H19	0.9300
			
C5—N1—C1	116.4 (4)	C7—C8—H8	107.9
C13—N2—C9	117.2 (4)	N2—C9—C10	122.1 (4)
C14—N3—C8	122.8 (3)	N2—C9—C8	114.5 (3)
C14—N3—H3N	118.6	C10—C9—C8	123.4 (3)
C8—N3—H3N	118.6	C9—C10—C11	119.0 (4)
N1—C1—C2	124.8 (5)	C9—C10—H10	120.5
N1—C1—H1	117.6	C11—C10—H10	120.5
C2—C1—H1	117.6	C12—C11—C10	119.9 (4)
C1—C2—C3	118.4 (5)	C12—C11—H11A	120.0
C1—C2—H2	120.8	C10—C11—H11A	120.0
C3—C2—H2	120.8	C13—C12—C11	117.2 (4)
C2—C3—C4	118.8 (4)	C13—C12—H12	121.4
C2—C3—H3	120.6	C11—C12—H12	121.4
C4—C3—H3	120.6	N2—C13—C12	124.5 (4)
C3—C4—C5	119.0 (4)	N2—C13—H13	117.7
C3—C4—H4	120.5	C12—C13—H13	117.7
C5—C4—H4	120.5	C19—C14—N3	122.5 (3)
N1—C5—C4	122.5 (4)	C19—C14—C15	118.6 (3)
N1—C5—C6	116.5 (4)	N3—C14—C15	118.9 (3)
C4—C5—C6	121.0 (4)	C16—C15—C14	120.3 (3)
O1—C6—C5	119.8 (4)	C16—C15—H15	119.9
O1—C6—C7	120.8 (4)	C14—C15—H15	119.9
C5—C6—C7	119.5 (3)	C15—C16—C17	121.9 (4)
C6—C7—C8	111.9 (3)	C15—C16—H16	119.0
C6—C7—H7A	109.2	C17—C16—H16	119.0
C8—C7—H7A	109.2	C18—C17—C16	118.1 (4)
C6—C7—H7B	109.2	C18—C17—H17	120.9
C8—C7—H7B	109.2	C16—C17—H17	120.9
H7A—C7—H7B	107.9	C17—C18—C19	121.5 (4)
N3—C8—C9	114.0 (3)	C17—C18—H18	119.3
N3—C8—C7	108.5 (3)	C19—C18—H18	119.3
C9—C8—C7	110.4 (3)	C14—C19—C18	119.6 (4)
N3—C8—H8	107.9	C14—C19—H19	120.2
C9—C8—H8	107.9	C18—C19—H19	120.2
			
C5—N1—C1—C2	−1.0 (7)	N3—C8—C9—N2	−157.1 (3)
N1—C1—C2—C3	0.7 (8)	C7—C8—C9—N2	80.3 (4)
C1—C2—C3—C4	0.0 (7)	N3—C8—C9—C10	24.8 (5)
C2—C3—C4—C5	−0.3 (7)	C7—C8—C9—C10	−97.7 (4)
C1—N1—C5—C4	0.7 (6)	N2—C9—C10—C11	−0.2 (6)
C1—N1—C5—C6	−178.4 (4)	C8—C9—C10—C11	177.7 (4)
C3—C4—C5—N1	−0.1 (6)	C9—C10—C11—C12	−0.8 (7)
C3—C4—C5—C6	179.0 (4)	C10—C11—C12—C13	1.3 (7)
N1—C5—C6—O1	167.1 (4)	C9—N2—C13—C12	0.0 (6)
C4—C5—C6—O1	−12.0 (6)	C11—C12—C13—N2	−0.9 (7)
N1—C5—C6—C7	−12.3 (5)	C8—N3—C14—C19	6.6 (5)
C4—C5—C6—C7	168.6 (4)	C8—N3—C14—C15	−175.7 (3)
O1—C6—C7—C8	51.2 (5)	C19—C14—C15—C16	1.5 (6)
C5—C6—C7—C8	−129.4 (3)	N3—C14—C15—C16	−176.3 (4)
C14—N3—C8—C9	67.7 (4)	C14—C15—C16—C17	−1.0 (6)
C14—N3—C8—C7	−168.8 (3)	C15—C16—C17—C18	0.0 (6)
C6—C7—C8—N3	69.9 (4)	C16—C17—C18—C19	0.6 (6)
C6—C7—C8—C9	−164.4 (3)	N3—C14—C19—C18	176.7 (4)
C13—N2—C9—C10	0.6 (5)	C15—C14—C19—C18	−0.9 (5)
C13—N2—C9—C8	−177.4 (3)	C17—C18—C19—C14	−0.1 (6)

### Hydrogen-bond geometry (Å, °)

**Table:**

*D*—H···*A*	*D*—H	H···*A*	*D*···*A*	*D*—H···*A*
N3—H3N···N2^i^	0.86	2.35	3.191 (4)	164
C10—H10···O1^i^	0.93	2.62	3.398 (5)	141
C3—H3···Cg^ii^	0.93	2.77	3.548 (5)	142

Symmetry codes: (i) −*x*+1, *y*−1/2, −*z*+3/2; (ii) *x*+1/2, −*y*+1/2, −*z*+1.
